# Visual Impairment and Risk of Dementia in 2 Population-Based Prospective Cohorts: UK Biobank and EPIC-Norfolk

**DOI:** 10.1093/gerona/glab325

**Published:** 2021-10-27

**Authors:** Thomas J Littlejohns, Shabina Hayat, Robert Luben, Carol Brayne, Megan Conroy, Paul J Foster, Anthony P Khawaja, Elżbieta Kuźma

**Affiliations:** 1 Nuffield Department of Population Health, University of Oxford, Oxford, UK; 2 Department of Public Health and Primary Care, University of Cambridge, Cambridge, UK; 3 NIHR Biomedical Research Centre at Moorfields Eye Hospital and UCL Institute of Ophthalmology, London, UK; 4 MRC Epidemiology Unit, University of Cambridge, Cambridge, UK; 5 Albertinen-Haus Centre for Geriatrics and Gerontology, University of Hamburg, Hamburg, Germany

**Keywords:** Epidemiology, Longitudinal, Prevention, Visual acuity

## Abstract

Visual impairment has emerged as a potential modifiable risk factor for dementia. However, there is a lack of large studies with objective measures of vision and with more than 10 years of follow-up. We investigated whether visual impairment is associated with an increased risk of incident dementia in UK Biobank and European Prospective Investigation into Cancer in Norfolk (EPIC-Norfolk). In both cohorts, visual acuity was measured using a “logarithm of the minimum angle of resolution” (LogMAR) chart and categorized as no (≤0.30 LogMAR), mild (>0.3 to ≤0.50 LogMAR), and moderate to severe (>0.50 LogMAR) impairment. Dementia was ascertained through linkage to electronic medical records. After restricting to those aged ≥60 years, without prevalent dementia and with eye measures available, the analytic samples consisted of 62 206 UK Biobank and 7 337 EPIC-Norfolk participants, respectively. In UK Biobank and EPIC-Norfolk, respectively, 1 113 and 517 participants developed dementia over 11 and 15 years of follow-up. Using multivariable Cox proportional-hazards models, the hazard ratios for mild and moderate to severe visual impairment were 1.26 (95% confidence interval [CI]: 0.92–1.72) and 2.16 (95% CI: 1.37–3.40), in UK Biobank, and 1.05 (95% CI: 0.72–1.53) and 1.93 (95% CI: 1.05–3.56) in EPIC-Norfolk, compared to no visual impairment. When excluding participants censored within 5 years of follow-up or with prevalent poor or fair self-reported health, the direction of the associations remained similar for moderate impairment but was not statistically significant. Our findings suggest visual impairment might be a promising target for dementia prevention; however, the possibility of reverse causation cannot be excluded.

Visual acuity is the ability to see clearly and is typically used to assess visual impairment, which can range from mild impairment to complete blindness (1). An estimated 440 million individuals worldwide live with visual impairment, with the prevalence of blindness projected to triple between 2020 and 2050 (2). However, visual impairment is often preventable or treatable through eye care programs, surgery, and corrective lenses (3). Recently, sensory impairments have emerged as potential risk factors for dementia, with the Lancet 2017 and 2020 Commissions on dementia prevention identifying hearing loss as a key modifiable risk factor (4,5). Visual impairment could increase dementia risk through mechanisms similar to hearing loss, such as the reallocation of cognitive resources to handle increased perceptual demands, or mediation through depression, social isolation, and physical inactivity (6–9).

A recent meta-analysis found that visual impairment was associated with a 47% increased risk of dementia when pooling data from 14 prospective studies (10). Despite promising findings for visual impairment as a target for dementia prevention, there is a lack of studies that combine a large sample size with a long follow-up period and an exposure ascertained using distance visual acuity. This is the clinical standard and underpins international taxonomies of visual impairment (11). Interpreting previous findings is further complicated by the heterogeneity of different study designs, methodological approaches, and exposure/outcome definitions. This could be addressed by replicating analyses in different populations, with similar exposure, covariate, and outcome ascertainment.

To address these limitations, we investigated the association between objectively measured visual impairment and the risk of incident dementia in 2 large population-based cohorts over 11 and 15 years of follow-up, respectively. We hypothesized that visual impairment would be associated with an increased risk of developing dementia compared to no visual impairment. We also hypothesized that there would be a dose–response effect, with increasing severity of visual impairment associated with greater dementia risk.

## Method

### UK Biobank

UK Biobank is a population-based prospective cohort study that recruited 503 317 women and men aged 40–69 from England, Scotland, and Wales between 2006 and 2010 (5.5% response rate) (12,13). At baseline, all participants provided electronic signed consent, answered questions on sociodemographic, lifestyle, and health-related factors, and completed a range of physical examinations. Eye measures were incorporated into the physical examinations at baseline assessment between 2009 and 2010 and were completed by approximately 117 252 participants. A further 16 016 participants who did not undergo the eye examination at baseline had eye measures collected during a repeat of baseline assessment between 2012 and 2013 (14). For the current study, date of first eye examination is defined as “baseline,” whether 2009–2010 or 2012–2013. UK Biobank received ethical approval from the National Health Service North West Centre for Research Ethics Committee (Ref: 11/NW/0382).

### European Prospective Investigation into Cancer in Norfolk

The European Prospective Investigation into Cancer in Norfolk (EPIC-Norfolk) is a population-based prospective cohort study of 25 639 women and men aged 40–79 years recruited between 1993 and 1997 (33% response rate) (15,16). Additional participants also joined the study at follow-up waves. Eye measures were introduced as part of a third health examination (EPIC-Norfolk 3—baseline for current study) between 2006 and 2011, including data from a pilot phase 2004–2006 (15,17). At examination, all participants provided written informed consent and completed a questionnaire on sociodemographic, lifestyle, and health-related factors. Ethical approval for EPIC-Norfolk core study was provided by the Norwich District Health Authority ethics committee (Rec Ref: 98NC01). EPIC-Norfolk 3 was approved by the Norfolk Local Research Ethics Committee (05/Q0101/191) and East Norfolk and Waveney National Health Service (NHS) Research Governance Committee (2005EC07L).

### Visual Function

The eye examinations in UK Biobank and EPIC-Norfolk included visual acuity, the most common clinical measurement of visual function. Visual acuity was measured in both eyes using “logarithm of the minimum angle of resolution” (LogMAR) characters (Precision Vision, LaSalle, IL), displayed on a computer screen in UK Biobank and on a light box in EPIC-Norfolk, both under standard illumination (17,18). The test in both cohorts was carried out with participants wearing usual, available, correction at 4 m, or at 1 m if participants were unable to read any letters. Participants were asked to read each letter from the end of each line going from top to bottom, until hesitation. In UK Biobank the test was terminated when ≥2 letters were incorrect. In EPIC-Norfolk the test was terminated when the participant was able to read ≤3 letters on a line and testing was repeated using pinhole-correction if participants were unable to read 3 letters on the 0.3 line. Standard letter by letter scoring was used to derive LogMAR visual acuity.

### Dementia

In UK Biobank, dementia status was recorded using hospital inpatient records obtained from Hospital Episode Statistics for England, Scottish Morbidity Record for Scotland, and Patient Episode Database for Wales as well as death registry records obtained from NHS Digital for England and Wales and Information and Statistics Division for Scotland. In EPIC-Norfolk, dementia was ascertained using hospital inpatient records obtained from Hospital Episode Statistics, death registry records as well as the following mental health care data sets which capture information on individuals in contact with mental health services and memory clinics; Mental Health Minimum Data Set, Mental Health and Learning Disabilities Data Set, and the Mental Health Services Data Set. All diagnoses were recorded using the International Classification of Diseases (ICD) coding system (see Supplementary Table 1 for list of ICD codes).

### Covariates

In both cohorts, Townsend deprivation score was used as an indicator of material deprivation and was assigned to each participant corresponding to the output area of their residential postcode at recruitment (19). Educational qualifications, ethnicity, smoking status, alcohol consumption, diabetes, and cardiovascular disease were collected via paper questionnaire in EPIC-Norfolk. The same variables were collected via the touch screen questionnaire in UK Biobank, except diabetes and cardiovascular disease, which were captured during a verbal interview conducted by a trained nurse. In both cohorts, body mass index (kg/m^2^) was derived from weight (kg) using scales and standing height (m) measured during the physical examinations (see Supplementary Table 2 for more information on covariate collection). In UK Biobank, the covariates were collected at both baseline (2009–2010) and repeat assessment (2012–2013). Covariates collected at the time of first eye measure were used in all analyses.

### Statistical Analysis

Person-years were calculated from the date of visual acuity measure until the first incident diagnosis of dementia, date of death, date lost-to follow-up, or end of follow-up, whichever came first. End of follow-up was based on the last possible date of electronic medical record availability. For UK Biobank this was November 30, 2020 for England, October 31, 2020 for Scotland, February 28, 2018 for Wales; for EPIC-Norfolk this was March 31, 2019. Cox proportional-hazards models were used to assess the association between visual impairment and risk of incident dementia. Visual impairment was categorized using the World Health Organization classification based on visual acuity in the better eye of “no impairment” (≤0.30 LogMAR), “mild impairment” (>0.3 to ≤0.50 LogMAR), and “moderate to severe impairment” (>0.50 LogMAR) (20). All models were assessed for the proportionality of hazards assumption using Schoenfeld residuals. In basic adjusted models we controlled for age in years, sex, ethnicity (White, non-White), and educational qualifications (no qualifications, lower secondary [ie, CSE/O-Level/GCSE or equivalent], upper secondary [ie, AS/A-Level or equivalent], higher education, or other equivalent professional qualification). In fully adjusted models we additionally controlled for socioeconomic status using Townsend deprivation score (quintiles), smoking status (never, former, current), alcohol intake (never, former, current), body mass index (<25, ≥25 to <30, ≥30 kg/m^2^), diabetes (no, yes), and cardiovascular disease (no, yes). Multiple imputation by chained equations with 100 imputations was used to impute missing values and values where participants responded “prefer not to answer” or “do not know,” for any covariates. The main exposure, outcome, and covariates were entered into the imputation model.

In a sensitivity analysis, the main models were repeated using a Fine–Gray subdistribution hazard model with death considered as a competing event (21).

Two separate sensitivity analyses were performed to explore reverse causation due to preclinical dementia potentially influencing visual and other health-related factors. These included (i) excluding participants with less than 5 years of follow-up and (ii) excluding participants who reported their health as poor or fair at baseline assessment.

In secondary analyses, the main analysis was repeated with visual acuity entered as a continuous variable to investigate the association with incident dementia per 0.10 LogMAR unit, which is equivalent to 5 letters or 1 line on a LogMAR chart. We also repeated the main model using a complete case analysis to investigate whether the results differed compared to using multiple imputation. Stratification by age was performed in UK Biobank to investigate effect modification by late-middle age (60–64 years) or early-older age (≥65 years). This analyses was not performed in EPIC-Norfolk due to the older age structure of the cohort. Furthermore, because the UK Biobank outcome adjudication working group’s recommended definition of dementia includes additional ICD codes compared to EPIC-Norfolk (Supplementary Table 1) (22), the UK Biobank analyses were repeated with the same ICD codes used to define dementia as EPIC-Norfolk. Due to the availability of genetic data in UK Biobank, the main analyses were repeated with additional adjustment for apolipoprotein E-ε4 carrier status (absence of ε4 alleles, presence of 1 or 2 ε4 alleles), a strong risk factor for dementia (23,24).

All *p* values were 2-sided, and the type I error rate for statistical significance was set at .05. Analyses were performed using Stata SE version 15.1 (StataCorp LLC, College Station, TX).

## Results

**Figure 1.**
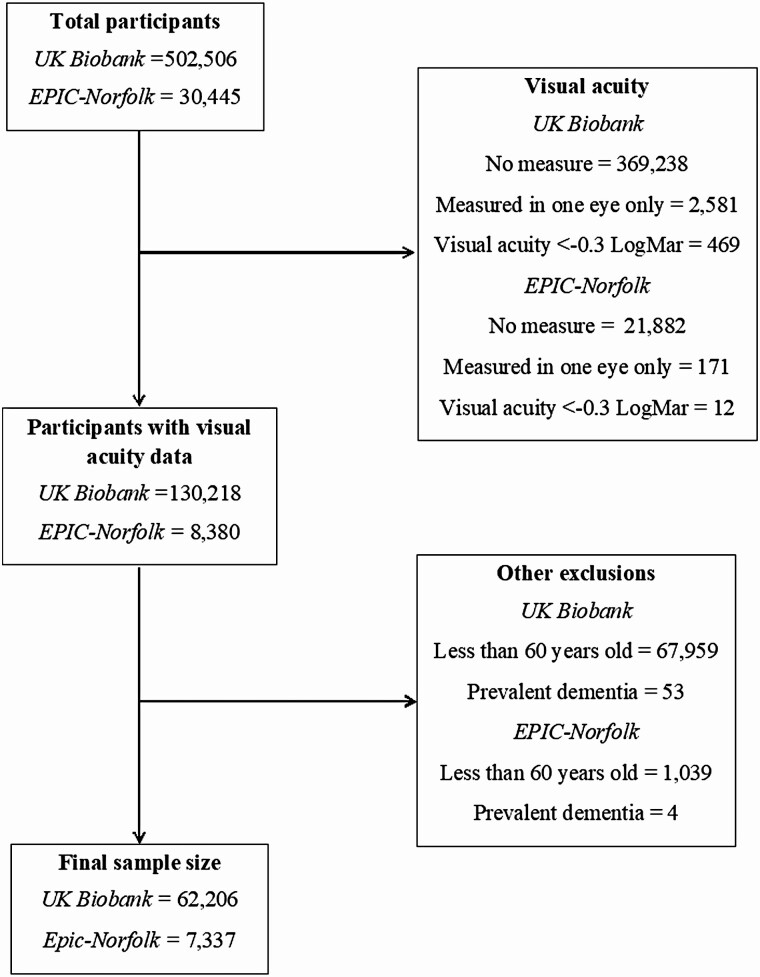
Flow chart for final analytic sample sizes in UK Biobank and European Prospective Investigation into Cancer in Norfolk (EPIC-Norfolk).

Of 502 506 UK Biobank and 30 445 EPIC-Norfolk participants, 130 218 and 8 380 participants had complete visual acuity data, respectively. After excluding participants less than 60 years old at baseline or with prevalent dementia, the final sample size in UK Biobank and EPIC-Norfolk was 62 206 and 7 337 participants, respectively (Figure 1).

In UK Biobank, a total of 1 113 newly recorded hospital inpatient dementia cases or dementia-registered deaths were captured over 616 117 person-years of follow-up (mean = 9.9 years, standard deviation = 1.8), whereas in EPIC-Norfolk, a total of 517 incident dementia cases were captured over 68 709 person-years of follow-up (mean = 9.4 years, standard deviation = 2.5). In UK Biobank and EPIC-Norfolk, respectively, 1 549 (2.5%) and 216 (2.9%) participants had mild visual impairment, and 463 (0.7%) and 49 (0.7%) participants had moderate to severe visual impairment. Baseline characteristics by visual impairment status for both cohorts are provided in Table 1.

**Table 1.** Baseline Characteristics of 62 206 UK Biobank and 7 337 EPIC-Norfolk Participants by Visual Impairment Status

	UK Biobank			EPIC-Norfolk		
	Visual Impairment (LogMAR)			Visual Impairment (LogMAR)		
Characteristic, *N* (%)	None (≤0.3) *N* = 60 194	Mild (>0.3 to ≤0.5) *N* = 1 549	Moderate to severe (>0.5) *N* = 463	None (≤0.3) *N* = 7 072	Mild (>0.3 to ≤0.5) *N* = 216	Moderate to severe (>0.5) *N* = 49
Age in years, mean (*SD*)	64.5 (3.1)	65.0 (3.1)	64.7 (3.1)	70.2 (6.9)	76.0 (7.2)	76.1 (7.8)
Women	31 217 (51.9)	812 (52.4)	239 (52.6)	3 813 (53.9)	126 (58.3)	27 (55.1)
Townsend deprivation score, quintiles						
** **1 (least deprived)	12 143 (20.2)	249 (16.1)	65 (14.0)	1 806 (25.5)	48 (22.2)	10 (20.4)
** **2	12 100 (20.1)	238 (15.4)	76 (16.4)	1 453 (20.5)	41 (19.0)	10 (20.4)
** **3	12 049 (20.0)	299 (19.3)	81 (17.5)	1 408 (19.9)	37 (17.1)	7 (14.3)
** **4	12 015 (20.0)	316 (20.4)	103 (22.3)	1 218 (17.2)	48 (22.2)	10 (20.4)
** **5 (most deprived)	11 845 (19.7)	445 (28.7)	138 (29.8)	1 168 (16.5)	42 (19.4)	12 (24.5)
** **Missing	42 (0.1)	2 (0.1)	0 (0)	19 (0.3)	0 (0)	0 (0)
Education						
** **No qualifications	12 656 (21.0)	483 (31.2)	143 (30.9)	1 935 (27.4)	68 (31.5)	29 (59.2)
** **Lower secondary	9 536 (15.8)	232 (15.0)	79 (17.1)	804 (11.4)	26 (12.0)	1 (2.0)
** **Upper secondary	2 907 (4.8)	75 (4.8)	17 (3.7)	3 159 (44.7)	94 (43.5)	12 (24.5)
** **Higher education or other professional qualification or equivalent	34 373 (57.1)	716 (46.2)	213 (46.0)	1 173 (16.6)	27 (12.5)	7 (14.3)
** **Missing/prefer not to answer	722 (1.2)	43 (2.8)	11 (2.4)	1 (0)	1 (0.5)	0 (0)
Ethnic background						
** **White	56 936 (94.6)	1 369 (88.4)	402 (86.8)	7 033 (99.4)	214 (99.1)	47 (95.9)
** **Non-White	2 885 (4.8)	153 (9.9)	54 (11.7)	20 (0.3)	0 (0.0)	1 (2.0)
** **Missing/prefer not to answer/do not know	373 (0.6)	27 (1.7)	7 (1.5)	19 (0.3)	2 (0.9)	1 (2.0)
Alcohol intake frequency						
** **Never	2 821 (4.7)	118 (7.6)	42 (9.1)	358 (5.1)	7 (3.2)	3 (6.1)
** **Former	2 177 (3.6)	76 (4.9)	12 (2.6)	790 (11.2)	29 (13.4)	5 (10.2)
** **Current	55 045 (91.5)	1 339 (86.4)	405 (87.5)	5 551 (78.5)	165 (76.4)	33 (67.35)
** **Missing/prefer not to answer	151 (0.3)	16 (1.0)	6 (0.9)	373 (5.3)	15 (6.9)	8 (16.3)
Smoking status						
** **Never	30 919 (51.4)	787 (50.8)	240 (51.8)	3 410 (48.2)	99 (45.8)	15 (30.6)
** **Former	24 681 (41.0)	588 (38.0)	173 (37.4)	3 294 (46.6)	102 (47.2)	28 (57.1)
** **Current	4 236 (7.0)	152 (9.8)	44 (9.5)	264 (3.7)	11 (5.1)	3 (6.1)
** **Missing/prefer not to answer	358 (0.6)	22 (1.4)	6 (1.3)	104 (1.5)	4 (1.9)	3 (6.1)
BMI						
** **<25	18 712 (31.1)	448 (28.9)	150 (32.4)	2 444 (34.6)	81 (37.5)	21 (42.9)
** **25–29.9	26 694 (44.4)	666 (43.0)	196 (42.3)	3 258 (46.1)	95 (44.0)	22 (44.9)
** **≥30	14 517 (24.1)	410 (26.5)	108 (23.3)	1 358 (19.2)	38 (17.6)	6 (12.2)
** **Missing	271 (0.5)	176 (11.4)	50 (10.8)	12 (0.2)	2 (1.0)	0 (0)
Diabetes	4 425 (7.4)	142 (9.2)	40 (8.6)	233 (3.3)	6 (2.8)	0 (0.0)
Cardiovascular disease	6 150 (10.2)	176 (11.4)	50 (10.8)	351 (5.0)	19 (8.8)	10 (20.4)
Overall health rating						
** **Poor/fair	15 776 (26.2)	1 054 (68.0)	307 (66.3)	1 090 (15.4)	34 (15.7)	16 (32.7)
** **Good/excellent	44 130 (73.3)	479 (30.9)	150 (32.4)	5 825 (82.4)	178 (82.4)	29 (59.2)
** **Missing/prefer not to answer/do not know	288 (0.5)	16 (1.0)	6 (1.3)	157 (2.2)	4 (1.9)	4 (8.2)

*Notes*: BMI = body mass index; EPIC-Norfolk = European Prospective Investigation into Cancer in Norfolk; LogMAR = logarithm of the minimum angle of resolution; *SD* = standard deviation.

**Figure 2.**
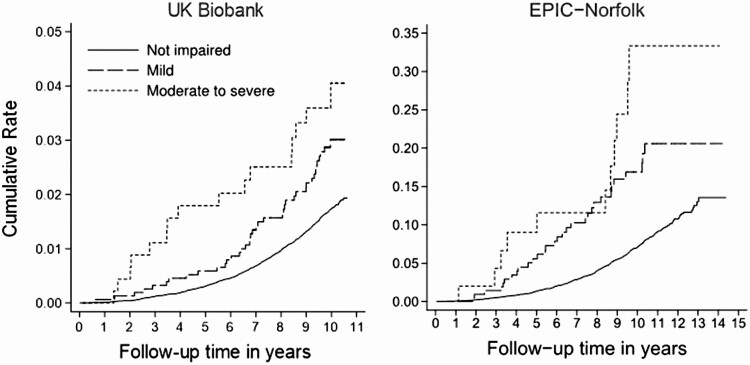
Cumulative hazard of dementia by visual impairment status.

A Nelson–Aalen cumulative hazards plot of dementia demonstrates clear differences in risk by visual impairment status after 1–2 years of follow-up in both cohorts (Figure 2). In basic adjusted models, the risk of dementia increased monotonically by visual impairment status in both cohorts, although the association between mild visual impairment and incident dementia was not statistically significant in either cohort (Table 2, see Supplementary Table 3 for effect of each additional covariate on the model estimates). The observed associations remained similar in fully adjusted models. Compared to those with no visual impairment, the hazard ratios (HRs) in UK Biobank and EPIC-Norfolk, respectively, were 1.26 (95% confidence interval [CI]: 0.92–1.72) and 1.05 (95% CI: 0.72–1.53) for mild impairment, and 2.16 (95% CI: 1.37–3.40) and 1.93 (95% CI: 1.05–3.56) for moderate to severe impairment. The direction of the associations remained similar when repeating the fully adjusted models with death as a competing event, although the effect sizes were attenuated for those with moderate impairment in both cohorts (Supplementary Table 4).

**Table 2.** Cox Proportional-Hazards Models for the Association Between Visual Impairment and Incident Dementia

		Visual Impairment (LogMAR)					
		None (≤0.3)		Mild (>0.3 to ≤0.5)		Moderate to Severe (>0.5)	
Cohort	Cases/Population	N	HR (95% CI)	N	HR (95% CI)	N	HR (95% CI)
UK Biobank							
Model A*	1 113/62 206	61 194	1 (reference)	1 549	1.32 (0.96–1.80)	463	2.17 (1.38–3.41)
Model B†	1 113/62 206	61 194	1 (reference)	1 549	1.26 (0.92–1.72)	463	2.16 (1.37–3.40)
EPIC-Norfolk							
Model A*	517/7 337	7 072	1 (reference)	216	1.10 (0.76–1.59)	49	1.86 (1.02–3.39)
Model B†	517/7 337	7072	1 (reference)	216	1.05 (0.72–1.53)	49	1.93 (1.05–3.56)

*Notes*: CI = confidence interval; EPIC-Norfolk = European Prospective Investigation into Cancer in Norfolk; HR = hazard ratio; LogMAR = logarithm of the minimum angle of resolution.

*Adjusted for age, sex, ethnicity, and education.

^†^Adjusted for age, sex, ethnicity, education, Townsend deprivation score, alcohol, smoking, body mass index, diabetes, and cardiovascular disease.

In sensitivity analyses, the main findings were attenuated in both cohorts when excluding participants with less than 5 years follow-up (Table 3). For instance, the HRs for moderate to severe impairment were 1.51 (95% CI: 0.83–2.74) and 1.51 (95% CI: 0.71–3.24) in UK Biobank and EPIC-Norfolk, respectively, compared to no impairment. After excluding participants with poor or fair self-reported health in UK Biobank, compared to no impairment, the associations remained similar to the main findings for mild impairment (HR = 1.44, 95% CI: 0.97–2.16) but was weaker for moderate to severe impairment (HR = 1.29, 95% CI: 0.58–2.88; Table 3). Whereas after excluding participants with poor or fair self-reported health in EPIC-Norfolk, the strength of the association for moderate to severe impairment was similar to the main findings, albeit attenuated (HR = 2.01, 95% CI: 0.89–4.57), compared to no impairment.

**Table 3.** Cox Proportional-Hazards Models for the Association of Visual Impairment and Incident Dementia Accounting for Reverse Causation

		Visual Impairment (LogMAR)					
		None (≤0.3)		Mild (>0.3 to ≤0.5)		Moderate to Severe (>0.5)	
Cohort	Cases/Population	N	HR (95% CI)	N	HR (95% CI)*	N	HR (95% CI)*
Excluding participants with <5 years follow-up							
UK Biobank	915/60 384	58 466	1 (reference)	1 489	1.18 (0.83–1.68)	429	1.51 (0.83–2.74)
EPIC-Norfolk	411/6 827	6 609	1 (reference)	180	0.82 (0.51–1.30)	38	1.51 (0.71–3.24)
Excluding participants with poor/fair self-reported health							
UK Biobank	627/45 801	44 418	1 (reference)	1 070	1.44 (0.97–2.16)	313	1.29 (0.58–2.88)
EPIC-Norfolk	379/6 032	5 825	1 (reference)	178	1.00 (0.65–1.55)	29	2.01 (0.89–4.57)

*Notes*: CI = confidence interval; EPIC-Norfolk = European Prospective Investigation into Cancer in Norfolk; HR = hazard ratio; LogMAR = logarithm of the minimum angle of resolution.

*Adjusted for age, sex, ethnicity, education, Townsend deprivation score, alcohol, smoking, body mass index, diabetes, and cardiovascular disease.

In fully adjusted models, the risk of dementia increased by 15% (HR = 1.15, 95% CI: 1.11–1.19) and 6% (HR = 1.06, 95% CI: 1.01–1.13) per 0.1 increase in LogMAR in UK Biobank and EPIC-Norfolk, respectively. In complete cases analyses, the direction of the associations remained similar to the main findings, although the strength was attenuated, in particular for those with moderate visual impairment (Supplementary Table 5). In analyses stratified by age in UK Biobank, the associations were stronger in late-middle-aged participants (60–64 years) and weaker in early-older-aged participants (≥65 years, Supplementary Table 6). In UK Biobank, when restricting to the same ICD codes used to ascertain dementia as EPIC-Norfolk there were 959 incident cases, and the HRs were 1.23 (95% CI: 0.90–1.69) and 2.05 (95% CI: 1.29–3.28) for mild and moderate to severe impairment, respectively, compared to no impairment in a fully adjusted model. In UK Biobank, the findings remained similar when additionally adjusted for apolipoprotein E-ε4 carrier status (HR = 1.29, 95% CI: 0.93–1.78 and HR = 2.12, 95% CI: 1.35–3.35 for mild and moderate impairment, respectively).

## Discussion

In 2 large cohorts of middle- to older-aged women and men recruited from the general population, moderate to severe visual impairment was associated with double the risk of incident dementia compared to normal vision. Mild visual impairment was associated with approximately 25% increased risk of dementia in UK Biobank, although the association was not statistically significant. There was limited evidence for an association between mild visual impairment and dementia in EPIC-Norfolk. In both cohorts, the main findings were attenuated when excluding those with less than 5 years of follow-up or prevalent poor or fair self-reported health and therefore reverse causation cannot be ruled out.

Our main findings are consistent with results from a meta-analysis of 14 prospective studies by Shang and colleagues, which found an increased risk of dementia in those with visual impairment compared to no impairment (relative risk = 1.47, 95% CI: 1.36–1.60) (10). This included unpublished UK Biobank results of similar strength to the current study (relative risk = 1.78, 95% CI: 1.18–2.68), though not directly comparable to our findings due to the lack of detail on the study design and apparent differences in sample composition (eg, unpublished results had a bigger analytic sample but fewer dementia cases).

The meta-analysis included studies that used various methods to define visual impairment, such as self-report (25–28), medical records (29,30), and color vision (31). In UK Biobank and EPIC-Norfolk, a distance visual acuity test, the clinical standard for determined visual impairment, was used. To our knowledge, 3 previous population-based studies have used the same method to ascertain visual impairment (32–34). In 2 008 U.S.-based adults, mild visual impairment or worse was associated with a nonsignificant increased risk of dementia (HR = 1.26, 95% CI: 0.90–1.77) over 10 years follow-up (32). In 1 061 U.S.-based adults, mild visual impairment or worse was associated with an increased risk of dementia (HR = 2.14, 95% CI: 1.08–4.21) over 7 years follow-up (34). In 15 506 Hong Kong-based adults, a monotonic association was observed in relation to dementia risk for mild (HR = 1.56, 95% CI: 1.17–2.06), moderate (HR = 2.27, 95% CI: 1.68–3.06), and severe or worse (HR = 10.84, 95% CI: 6.60–17.81) impairment over 6 years follow-up (33).

Previous studies generally indicate that mild visual impairment is associated with an increased dementia risk. However, we observed a weak association between mild visual impairment and incident dementia in UK Biobank (HR = 1.26, 95% CI: 0.92–1.72) and a lack of association in EPIC-Norfolk (HR = 1.05, 95% CI: 0.72–1.53). It is possible that age modifies the associations between milder forms of visual impairment and dementia risk, as the strongest associations were observed in those who were 60–64 years at baseline compared to participants 65 years or older in UK Biobank, whereas the overall associations in the older EPIC-Norfolk population were weaker than the younger UK Biobank population. This is consistent with findings that certain risk factors, such as hearing impairment, are hypothesized to increase the likelihood of dementia primarily at midlife rather than late life (5). Alternatively, differential responses might exist across age groups, and more studies exploring the effect of age on associations between vision and dementia risk are necessary.

Another explanation is that the findings are driven by reverse causation. The long prodromal period of dementia can affect exposures measured several years prior to a clinical dementia diagnosis, which in turn can produce spurious associations in studies with short follow-up (35–37). The meta-analysis by Shang and colleagues found similar associations between visual impairment and incident dementia when restricting to studies with 10 or more years follow-up (HR = 1.53, 95% CI: 1.30–1.80) and less than 10 years follow-up (HR = 1.50, 95% CI: 1.23–1.83) (10). However, even in studies with longer follow-up periods, the overall associations could be driven by cases that develop within the first few years. In the current study, when excluding participants with less than 5 years follow-up, the direction of associations remained the same but were substantially weaker. Lee and colleagues found that an increased risk of dementia remained for those with moderate and severe, but not mild, visual impairment when excluding cases within 3 years of follow-up (33). Naël and colleagues found that mild and moderate near visual impairment were significantly associated with dementia risk before, but not after, 4 years of follow-up (38).

There are several other potential explanations for the observed associations between visual impairment and dementia. Visual impairment is related to a poorer quality of life, a decline in physical and functional activities, social isolation, and an increased risk of depression (6–9,39,40), and these factors could lead to an increased risk of dementia (5,38,41). Impaired visual processing could adversely affect cognitive functioning directly through various mechanisms, such as sensory deprivation, increased perceptual load, or information degradation (42–44). Alternatively, visual and cognitive impairment and dementia risk could be linked by a “common cause” (43). In this scenario, impaired visual acuity could represent a promising predictive marker for dementia risk rather than a target for prevention. Certain visual conditions have been previously proposed as biomarkers for dementia, such as retinal nerve fiber layer thinning, abnormal pupillary response, and contrast sensitivity (45,46). An additional noncausal explanation is potential detection bias, where individuals with visual impairment perform worse on visually based cognitive tests or have increased utilization of healthcare services.

In addition to visual impairment, studies have explored the link between specific eye diseases and risk of dementia, although the evidence is mixed. A meta-analysis of prospective studies found that cataracts and diabetic retinopathy, but not glaucoma or age-related macular degeneration, were associated with dementia risk (47). Similar associations have been observed in UK Biobank, although in contrast, age-related macular degeneration was weakly associated with dementia risk (48). The potential mechanisms underlying the associations between eye diseases and dementia are complex; for instance, they could increase the risk of dementia via visual impairment, be confounded by other factors (ie, diabetes in the case of diabetic retinopathy) or share similar neurodegenerative pathways as dementia (ie, glaucoma) (49). Due to this complexity, we investigated visual impairment independently of eye diseases in the current study. Studies which explore whether associations between certain eye diseases and dementia are mediated by visual impairment or driven by other factors are warranted.

Our study has several strengths. Both cohorts utilized a similar eye assessment protocol, measured the same covariates, and captured dementia using longitudinal hospital and death registry records. This enabled us to replicate the analysis using standardized criteria in 2 separate populations with different age structures and different population characteristics. Both studies captured dementia through ongoing linkage to cohort-wide electronic medical records minimizing loss to follow-up. Participants were assessed with habitual correction (ie, glasses or contact lenses) which should provide an accurate measure of usual day-to-day visual function.

Our study also has several limitations. Despite the large sample size in both cohorts, the proportion of individuals with moderate visual impairment was small. This limited the potential for additional analyses due to lack of statistical power, such as investigating whether the associations were mediated through other factors. As there was no information on how long participants have been visually impaired or repeat visual acuity measures we were unable to investigate whether the results were affected by time impaired or account for exposure change over time.

We did not investigate specific dementias, such as Alzheimer’s disease or vascular dementia, due to the poor positive predictive value of the hospital inpatient and death records for ascertaining subtypes (50). However, validation studies have found that these are reliable for ascertaining all-cause dementia, with a positive predictive value of 84.5% in UK Biobank when compared with expert clinical adjudication (50,51). Nevertheless, the hospital inpatient and death records are likely to capture dementia cases in the later stages. For instance, 1 study found that dementia cases originally diagnosed in primary care are captured in hospital records an average of 1.6 years later (52). A degree of misclassification bias is likely whereby dementia cases not captured in the available medical records are treated as controls, which could bias the effect sizes toward the null.

Both cohorts were volunteer-based, with strong evidence of a “healthy volunteer effect” in UK Biobank (13,15). A recent study found that similar exposure–outcome associations were observed in UK Biobank compared to a representative cohort for cause-specific deaths; nevertheless, selection bias could remain (53,54). Due to the observational design, a degree of residual confounding is likely and causality cannot be inferred.

Mild and moderate to severe visual impairment was monotonically associated with an increased likelihood of developing dementia. Visual impairment has a high prevalence, especially in middle-later life, but is often treatable or preventable and consequently could be a promising target for dementia prevention. However, further research is needed to establish whether visual impairment is a dementia risk factor, an early sign of dementia, or whether the age at onset of visual impairment or its duration plays a differential role.

## Funding

A.P.K. was funded by a UKRI Future Leaders Fellowship. E.K. was supported by the Nicolaus and Margrit Langbehn Foundation. The EPIC-Norfolk study (doi:10.22025/2019.10.105.00004) has received funding from the Medical Research Council (MR/N003284/1 and MC-UU_12015/1) and Cancer Research UK (C864/A14136). The funding sources had no role in the design and conduct of the study; collection, management, analysis, and interpretation of the data; preparation, review, or approval of the manuscript; and decision to submit the manuscript for publication.

## Conflict of Interest

A.P.K. has performed consultancy work for Aerie, Allergan, Google Health, Novartis, Reichert, Santen, and Thea. The other authors declare no conflict of interest.

## Supplementary Material

glab325_suppl_Supplementary_Materials
