# Supplementary material for: Visual Impairment and Risk of Dementia in 2 Population-Based Prospective Cohorts: UK Biobank and EPIC-Norfolk
Source: J Gerontol A Biol Sci Med Sci. 2021 Oct 27;77(4):697–704. doi: 10.1093/gerona/glab325 (PMC8974347; doi:10.1093/gerona/glab325)
Supplement: glab325_suppl_Supplementary_Materials [file glab325_suppl_supplementary_materials.pdf]

eTable 1 – International Classification of Disease codes used to ascertain dementia

eTable 2 – Measurement of covariates in UK Biobank and EPIC-Norfolk assessment centres

eTable 3 – Cox proportional-hazards models for the association between visual impairment and incident dementia with sequential adjustment for covariates

eTable 4 – Subdistribution hazard models for the association between visual impairment and incident dementia with death as a competing event

eTable 5 – Cox proportional-hazards models for the association between visual impairment and incident dementia without multiple imputation for covariates

eTable 6 – Cox-proportional-hazards models for the association between visual impairment and incident dementia stratified by age in UK Biobank

eTable 1 – International Classification of Disease codes used to ascertain dementia

Cohort	ICD-9	ICD-10
UK Biobank	331.0, 290.4, 331.1, 290.2, 290.3, 291.2, 294.1, 331.2, 331.5	F00, F00.0, F00.1, F00.2, F00.9, G30, G30.0, G30.1, G30.8, G30.9, F01, F01.0, F01.1, F01.2, F01.3, F01.8, F01.9, I67.3, F02.0, G31.0, A81.0, F02, F02.1, F02.2, F02.3, F02.4, F02.8, F03, F05.1, F10.6, G31.1, G31.8
EPIC-Norfolk	Not applicable	F00, F00.0, F00.1, F00.2, F00.9, G30, G30.0, G30.1, G30.8, G30.9, F01, F01.0, F01.1, F01.2, F01.8, F01.9, F02.0, G31.0, F02, F02.1, F02.2, F02.3, F02.8, F03, F05.1, G31.8

ICD, International Classification of Disease

eTable 2 – Measurement of covariates in UK Biobank and EPIC-Norfolk assessment centres

Covariate	Measurement in UK Biobank	Measurement in EPIC-Norfolk
Age	Derived from date of birth and date of attending assessment centre	Derived from date of birth and date of attending assessment centre
Sex	Acquired from central registry, updated by participant.	Baseline Health and Lifestyle questionnaire
Education	Touchscreen questionnaire<br>"Which of the following qualifications do you have? (you can select more than one)"	Baseline Health and Lifestyle questionnaire
Ethnicity	An amalgam of sequential branching questions asked during the initial assessment centre visit as part of the touchscreen questionnaire	Not applicable
Alcohol consumption	Touchscreen questionnaire<br>"About how often do you drink alcohol?"	Follow-up IV Health and Lifestyle questionnaire<br>'Did you drink alcohol in the last week'
Smoking	Touchscreen questionnaire<br>Derived from following questions;<br>Never/Current - "Do you smoke now?"<br>Past = "In the past, how often have you smoked tobacco?"	Follow-up IV Health and Lifestyle questionnaire<br>Derived from following questions;<br>Do you currently smoke cigarettes<br>If you have stopped smoking, how old were you when you gave up.<br>Current - "Do you smoke now?"<br>Never smoking – was ascertained from previous Health and Lifestyle questionnaire from baseline and second health check phases
BMI	Physical exam<br>Derived from height and weight	Physical exam<br>Derived from height and weight at the 3rd health examination
Diabetes	Verbal interview with a trained nurse	Medical history was ascertained with the question "Has a doctor ever told you that you have any of the following?" followed by a list of conditions that included heart attack, stroke, diabetes, depression requiring treatment, pulmonary disease, asthma and cancer<br>Ascertained from any previous Health and Lifestyle questionnaire
Cardiovascular disease	Verbal interview with a trained nurse	As with diabetes

Note. BMI, Body Mass Index

eTable 3 – Cox proportional-hazards models for the association between visual impairment and incident dementia with sequential adjustment for covariates

	Visual Impairment (LogMAR)		
	None (≤0.3)	Mild (>0.3-≤0.5)	Moderate to severe (>0.5)
Cohort	HR (95% CI)	HR (95% CI)	HR (95% CI)
**UK Biobank**			
Age and sex	1 (reference)	1.40 (1.02-1.91)	2.26 ((1.43-3.56)
+ Ethnicity and education	1 (reference)	1.32 (0.96-1.80)	2.17 (1.38-3.41)
+ Townsend deprivation score	1 (reference)	1.28 (0.94-1.75)	2.11 (1.34-3.32)
+ Alcohol and smoking	1 (reference)	1.25 (0.92-1.72)	2.12 (1.35-3.34)
+ Body mass index	1 (reference)	1.25 (0.92-1.71)	2.10 (1.33-3.31)
+ Diabetes and cardiovascular disease	1 (reference)	1.26 (0.92-1.72)	2.16 (1.37-3.40)
**EPIC-Norfolk**			
Age and sex	1 (reference)	1.10 (0.76-1.59)	1.86 (1.02-3.39)
+ Ethnicity and education	1 (reference)	1.09 (0.76-1.58)	1.86 (1.02-3.40)
+ Townsend deprivation score	1 (reference)	1.07 (0.74-1.55)	1.82 (0.99-3.32)
+ Alcohol and smoking	1 (reference)	1.07 (0.74-1.55)	1.79 (0.98-3.27)
+ Body mass index	1 (reference)	1.06 (0.73-1.54)	1.78 (0.97-3.26)
+ Diabetes and cardiovascular disease	1 (reference)	1.05 (0.72-1.53)	1.93 (1.05-3.56)

Note. CI, Confidence Interval, HR, Hazard Ratio, LogMAR, Logarithm of the Minimum Angle of Resolution

eTable 4 – Subdistribution hazard models for the association between visual impairment and incident dementia with death as a competing event

		Visual impairment (LogMAR)					
		None (≤0.3)		Mild (>0.3-≤0.5)		Moderate (>0.5)	
Cohort	Cases/Population	N	SHR (95% CI)*	N	SHR (95% CI)[a]	N	SHR (95% CI)[a]
UK Biobank	1,113/62,206	61,194	1 (reference)	1,549	1.25 (0.91-1.71)	463	2.01 (1.27-3.18)
EPIC-Norfolk	517/7,337	7072	1 (reference)	216	1.10 (0.74-1.63)	49	1.70 (0.85-3.40)

Note. CI, Confidence Interval, SHR, Hazard Ratio
[a] Adjusted for age, sex, ethnicity, education, Townsend deprivation score, alcohol, smoking, body mass index, diabetes and cardiovascular disease

eTable 5 – Cox proportional-hazards models for the association between visual impairment and incident dementia without multiple imputation for covariates

		Visual impairment (LogMAR)					
		None (≤0.3)		Mild (>0.3-≤0.5)		Moderate (>0.5)	
**Cohort**	**Cases/Population**	**N**	**HR (95% CI)***	**N**	**HR (95% CI)[a]**	**N**	**HR (95% CI)[a]**
**UK Biobank**	1,069/60,537	58,650	1 (reference)	1,452	1.23 (0.88-1.70)	435	1.70 (1.00-2.89)
**EPIC-Norfolk**	471/6,888	6,651	1 (reference)	197	1.00 (0.67-149)	40	1.73 (0.85-3.54)

Note. CI, Confidence Interval, HR, Hazard Ratio
[a] Adjusted for age, sex, ethnicity, education, Townsend deprivation score, alcohol, smoking, body mass index, diabetes and cardiovascular disease

eTable 6 – Cox-proportional-hazards models for the association between visual impairment and incident dementia stratified by age in UK Biobank

		Visual impairment (LogMAR)					
		None (≤0.3)		Mild (>0.3-≤0.5)		Moderate to severe (>0.5)	
**Age, years**	**Cases/Population**	**N**	**HR (95% CI)**	**N**	**HR (95% CI)[a]**	**N**	**HR (95% CI)[*]**
**60-64**	330/32,086	31,430	1 (reference)	705	1.52 (0.85-2.70)	236	3.62 (1.79-7.32)
**>65**	783/29,835	28,764	1 (reference)	844	1.20 (0.83-1.75)	247	1.76 (0.97-3.19)

Note. CI, Confidence Interval, HR, Hazard Ratio, LogMAR, Logarithm of the Minimum Angle of Resolution
[a] Adjusted for sex, ethnicity, education, Townsend deprivation score, alcohol, smoking, body mass index, diabetes and cardiovascular disease
